# Rare Abdominopelvic Actinomycosis Causing an Intestinal Band Obstruction and Mimicking an Ovarian Malignancy

**DOI:** 10.7759/cureus.2721

**Published:** 2018-05-31

**Authors:** Sakthivel Chinnakkulam Kandhasamy, Byshetty Rajendar, Ashok Kumar Sahoo, Rajesh Nachiappa Ganesh, Mangala Goneppanavar, Vishnu Prasad Nelamangala Ramakrishnaiah

**Affiliations:** 1 General Surgery, Jawaharlal Institute of Postgraduate Medical Education and Research (JIPMER), Puducherry, IND; 2 Department of Pathology, Jawaharlal Institute of Postgraduate Medical Education and Research (JIPMER), Puducherry, IND; 3 HPB Surgery, Jawaharlal Institute of Postgraduate Medical Education and Research (JIPMER), Puducherry, IND

**Keywords:** abdominopelvic actinomycosis, intestinal obstruction, ovarian malignancy, abdominopelvic actinomycosis, intestinal obstruction, ovarian malignancy, band obstruction

## Abstract

Actinomyces israelii, a commensal of the bronchial and gastrointestinal tracts, is responsible for the majority of actinomycostic infections in humans. Actinomycosis has widely varying clinical presentations ranging from asymptomatic states to infiltrative mass lesions that mimic malignant abdominopelvic disease. Described as one of the most misdiagnosed diseases, actinomycosis poses challenges to accurate preoperative diagnosis. A 67-year-old woman with no significant medical history presented with features of acute intestinal obstruction. Computed tomography revealed a terminal ileal stricture causing intestinal obstruction and a right ovarian mass lesion. On laparotomy, a granular mass (2×2 cm) at the base of the mesentery and a right ovarian hard nodular growth (3×3 cm) were found that were connected by a dense fibrotic band, causing ileal obstruction with a transitional zone that was 10 cm proximal to the ileocecal junction. The mesenteric granular mass was excised together with the dense fibrotic band, and a right salpingo-oophorectomy was also undertaken. On postoperative histopathological examination, band formations by dense inflammatory tissue with neutrophilic infiltration were observed; moreover, there were sulfur granules that showed a positive reaction on Periodic Acid Schiff staining. The resected ovarian parenchyma showed infiltration by bacterial colonies with Splendore-Hoeppli phenomenon and evoked dense neutrophilic infiltration. The postoperative period was uneventful, and the patient was placed on penicillin therapy for a year. Abdominopelvic actinomycosis should constitute part of the differential diagnosis when evaluating mass lesions, especially in elderly women with a history of intrauterine device (IUD) use.

## Introduction

Actinomycosis is a rare disease caused by the gram-positive anaerobic bacteria Actinomyces israelii, a commensal microrganism of the alimentary, respiratory, and urogenital systems. The sites most commonly affected by actinomycosis are cervical (50–65%) and abdominal (20%), followed by the chest (15%) [1]. Despite a decrease in the overall incidence over the years, a slight increase in incidence has been associated with predisposing factors such as prior surgery, trauma, and various endoscopic interventions [2]. In women, long-standing intrauterine device (IUD) use may render the patient susceptible to pathogenicity by this commensal organism, and infection often presents as a mass lesion that mimics malignancy and should be differentiated from other conditions. Given the variable presentation of actinomycosis, preoperative diagnosis is often difficult. A large number of cases are often diagnosed only upon final histopathological and bacteriological examination of the resected specimen. Herein, we present the case of a 67-year-old woman with features of acute intestinal obstruction caused by dense fibrotic bands as well as a right ovarian mass mimicking a malignancy. The final histopathological analysis suggested abdominopelvic actinomycosis.

## Case presentation

**Figure 1:**
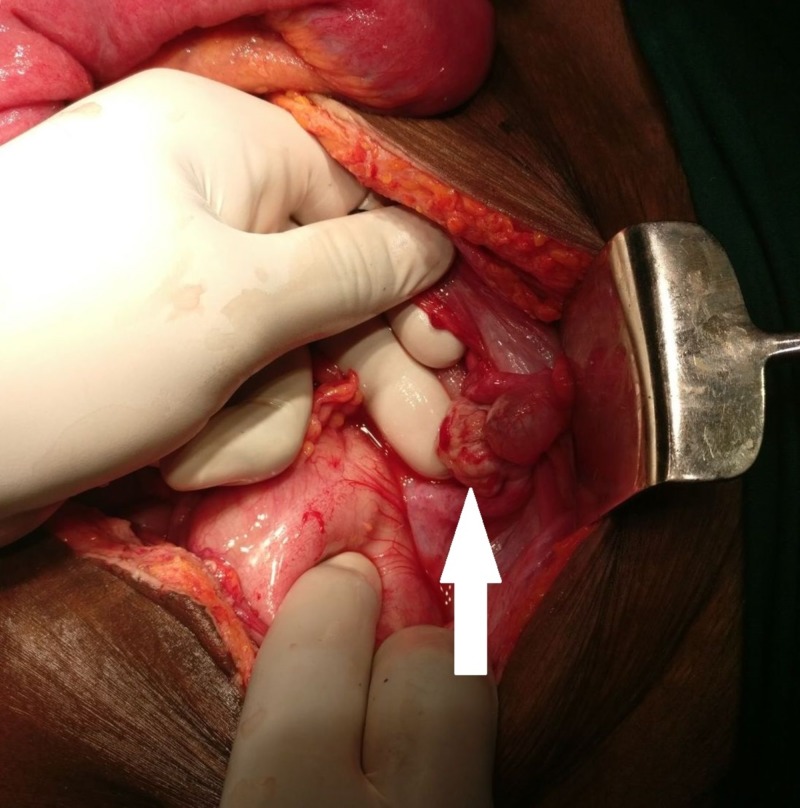
Right ovary with a hard nodular growth mimicking a malignancy (arrow)

A 67-year-old woman presented to the surgical emergency department with complaints of diffuse, colicky abdominal pain, abdominal distension, obstipation, and multiple episodes of bilious vomiting for six days. Moreover, the patient had a history of recent weight loss and loss of appetite. There was no history of vaginal discharge or IUD insertion or any significant past medical or surgical conditions. On examination, the patient was conscious, oriented, and had tachycardia with normal blood pressure. The abdomen was distended, with diffuse tenderness and guarding. On auscultation, bowel sounds were exaggerated. Abdominal X-ray showed multiple air-fluid levels with loops of distended small bowel. Contrast-enhanced computed tomography (CT) revealed a terminal ileal stricture close to the ileocecal junction together with proximal dilated, and distal collapsed, bowel loops, suggestive of intestinal obstruction. After optimal hemodynamic resuscitation, the patient underwent exploratory laparotomy under general anesthesia. Intraoperatively, we found a granular mass (2×2 cm) at the base of the mesentery and the right ovary with a hard nodular growth mimicking a malignancy (3×3 cm) (Figure 1).

**Figure 2:**
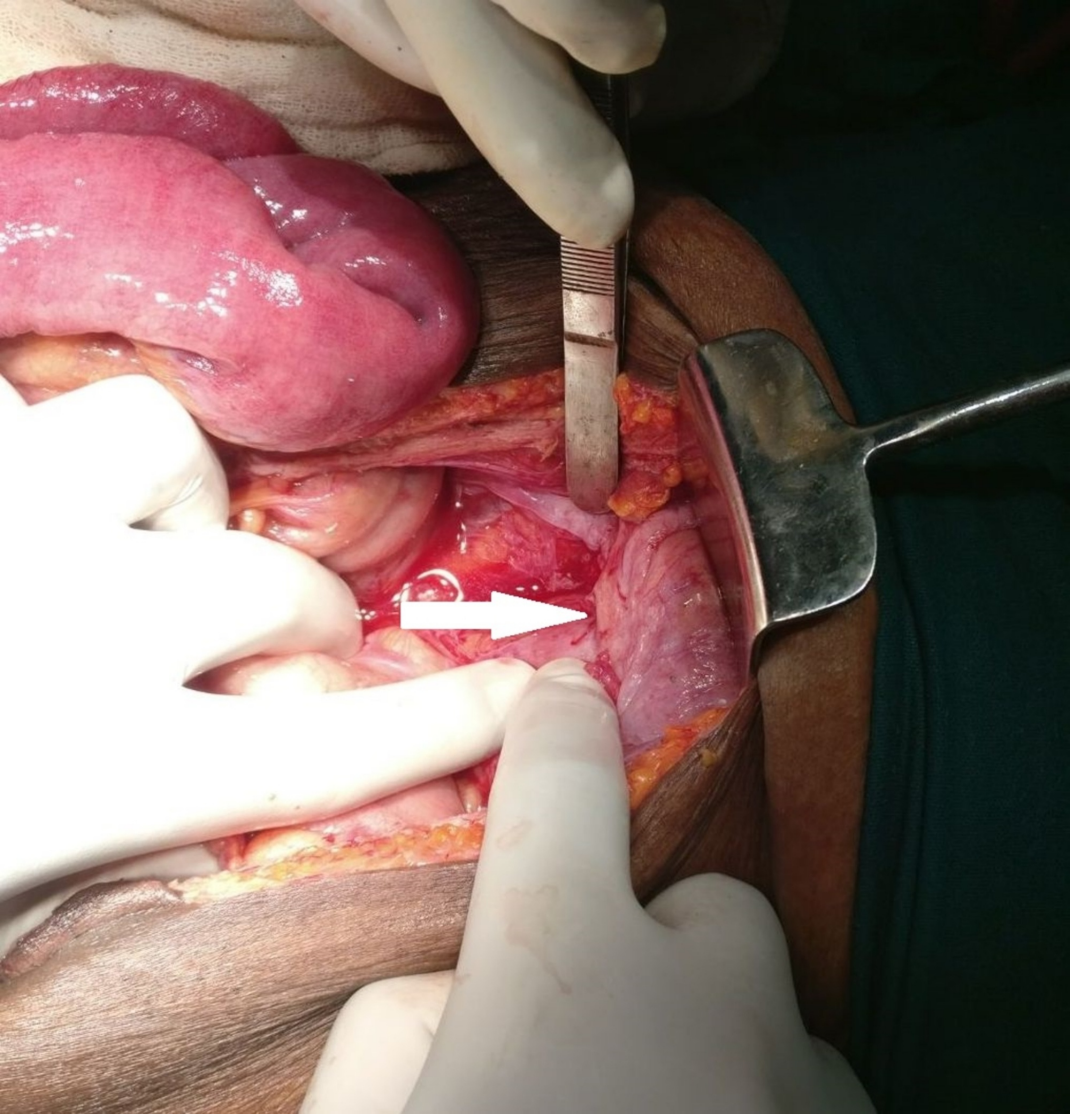
Dense fibrotic band causing small bowel obstruction (arrow)

A dense fibrotic band extended between the two masses, causing ileal obstruction, and a transitional zone was present 10 cm proximal to the ileocecal junction (Figure 2).

**Figure 3:**
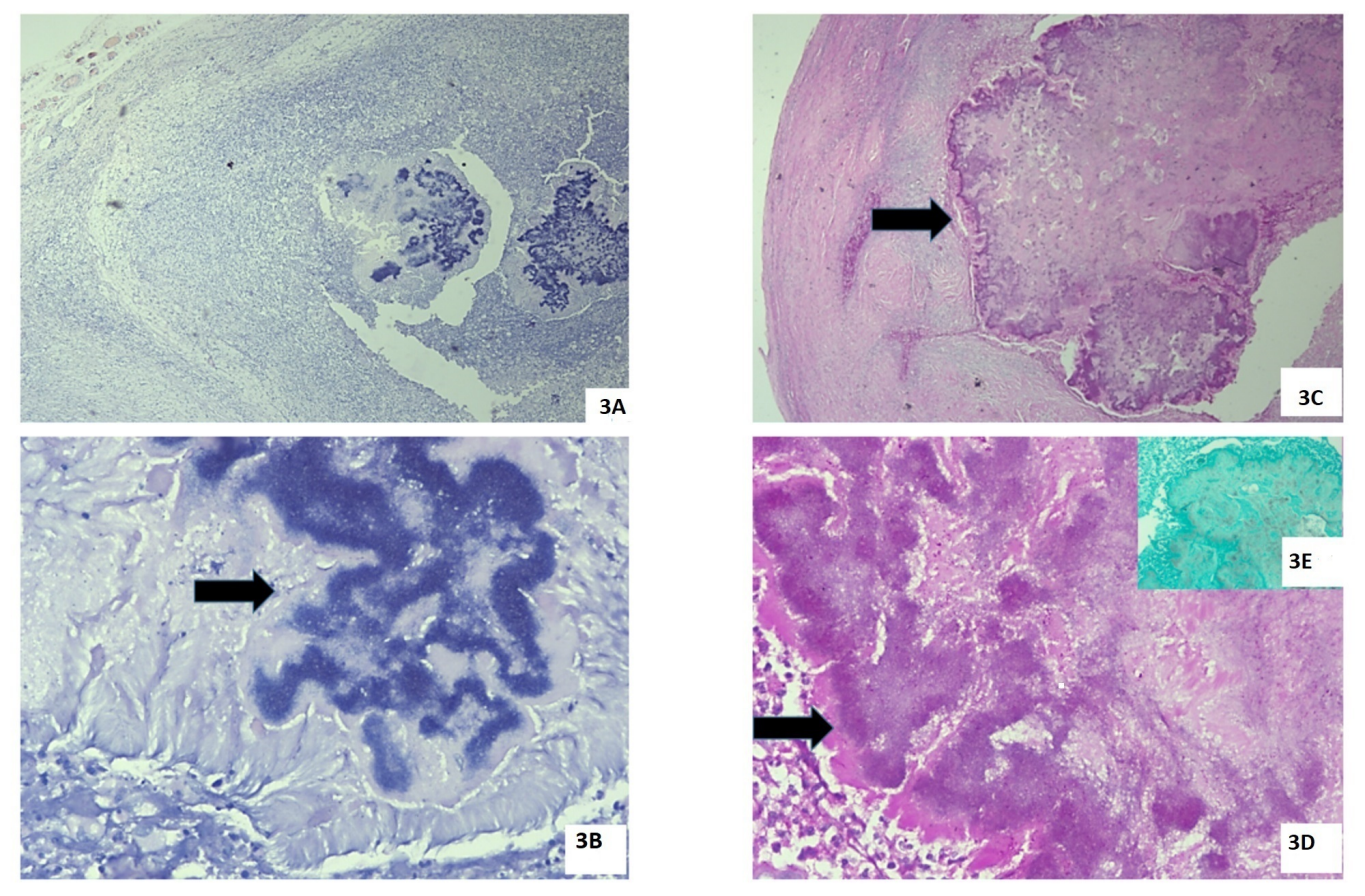
3A – This section shows the ovarian parenchyma infiltrated by bacterial colonies as well as the Splendore–Hoeppli phenomenon together with dense neutrophilic infiltration. Hematoxylin and eosin staining, ×100. 3B – This section shows a higher magnification of bacterial colonies (arrow) that is highlighted by the presence of granular bluish material surrounded by pink homogeneous immunoglobulin deposits (Splendore-Hoeppli phenomenon). Hematoxlyin and eosin staining, ×400. 3C – This section shows the same field stained by the Periodic Acid Schiff stain, demonstrating that no fungal organisms are present in the colony (arrow). Periodic Acid Schiff staining, ×100. 3D – This section shows the Splendore-Hoeppli phenomenon characterized by the bright magenta pink color on Period acid Schiff staining, ×400 (arrow). 3E (inset) – This section shows negative staining for fungal organisms on staining by the Gomori Methenamine Silver stain, ×100.

Both the mesenteric granular mass and the dense fibrotic band were excised, a right salphingo-oophorectomy was conducted, and resected specimens were sent for histopathological examination. On microscopic examination, the Actinomyces species was identified in the evaluated specimens, and a final diagnosis of abdominopelvic actinomycosis was made (Figures 3A-3E).

## Discussion

Actinomyces israelii is a saprophyte occurring commonly in the oral cavity, female urogenital system, and alimentary tract. Various predisposing factors could disrupt the normal barrier mechanism, leading to pathogenicity by commensals. The predisposing factors most often encountered include trauma, surgery, endoscopic procedures, chronic inflammatory processes, and immunocompromised status secondary to malignancy or diabetes mellitus or following a renal transplant [1].

Despite the widespread use of IUDs, which can lead to pelvic infections, abdominal/pelvic actinomycosis is a rare occurrence. Pelvic actinomycosis has been associated with IUD use in 80% of cases and is more frequent in those with an IUD usage duration of more than four years [1].

Clinical symptoms are often nonspecific, and the condition manifests with acute, subacute, or chronic presentations. Patients can present with acute abdominal pain, vomiting, constipation, and obstipation, as in the present case. Subacute conditions may present with symptoms of malabsorption, vague abdominal pain, loss of appetite, and weight loss. In chronic infection, actinomycosis may present with mass formations that mimic malignancy and strictures or fibrotic bands that lead to intestinal obstruction and perforation [2]. Some patients may show discharges containing sulfur granules, most often at sites where fine-needle aspiration was undertaken [1].

Abdominopelvic actinomycosis is difficult to diagnose preoperatively. Routine blood biochemistry and hematology rarely give a clue to this diagnosis. Ultrasonography may be used as an initial imaging technique that can exclude other common conditions. A plain abdominal radiograph may show features of intestinal obstruction and perforation that can aid symptomatic diagnosis. On contrast enhanced CT, bowel wall thickening and a homogenous enhancement pattern are detected more often. Some patients can have abdominal or pelvic masses that, often, may appear as a heterogeneous enhancement [3].

Abdominal actinomycosis can be included in the differential diagnosis when a CT scan shows bowel wall thickening, strictures, and a mass. This is especially noteworthy when there are non-specific signs in women with a history of IUD use [2]. Various chronic inflammatory conditions, such as Crohn’s disease and tuberculosis, mimic abdominal actinomycosis. Imaging findings suggestive of solid masses may induce confusion with malignancies of the alimentary or urogenital systems. Usually, actinomycosis shows up on imaging as a slow-attenuating solid mass than as thickened cystic lesions [3].

In the present case, CT showed stricture formation proximal to the ileocecal junction with dilation of proximal bowel loops and collapse of distal loops, which necessitated emergency surgery. In most cases, preoperative diagnosis is difficult as in the present case.

The final diagnosis was aided by histopathological examination of the surgical specimen. On examination, sulfur granules may be present, although it is not a pathognomic feature [4]; the granules are gram-positive and show a mycelial structure, often varying from 0.4 to 4 mm. These granules stain positive on staining with Periodic Acid Schiff and Grocott’s dye, and show a negative Kossa reaction; Nocardia and Streptomyces may show pseudoactinomycetic granules and demonstrate the opposite of this reaction. Sulfur granules can be seen in conditions such as nocardiosis, botryomycosis, eumycetoma, and streptomycosis [1]. In our patient, dense neutrophilic infiltration and band formation by fibroblasts, together with granule formation, led to the appearance of, seemingly, an ovarian malignancy [5]. During the inflammatory process, abscess formation and necrosis lead to an extensive inflammatory reaction that leads to mass formation.

## Conclusions

The primary establishment of a diagnosis of abdominopelvic actinomycosis is difficult. Clinical presentation is frequently variable, and presenting features may mimic a malignancy. Radiological imaging can reveal mass lesions that may be associated with a fluid collection, but it is difficult to distinguish among Crohn’s disease, tuberculosis, pelvic inflammatory disease, diverticulitis, and malignancy; abdominopelvic actinomycosis should be included as a differential diagnosis when evaluating these conditions. Despite diffuse disease, antibiotics either alone or in combination with surgery can cure the disease.

## References

[1] Pusiol T, Morichetti D, Pedrazzani P, Ricci F (2011). Abdominal-pelvic actinomycosis mimicking malignant neoplasm. Infect Dis Obstet Gynecol.

[2] Triantopoulou C, Van der Molen A, Van Es AC, Giannila M (2014). Abdominopelvic actinomycosis: spectrum of imaging findings and common mimickers. Acta Radiol Short Rep.

[3] Garner JP, Macdonald M, Kumar PK (2007). Abdominal actinomycosis. Int J Surg.

[4] Sánchez Sánchez M, Albareda Landívar J, Cano Serrano AM, García Rubio MJ, Marcello Fernández ME (2017). Abdominopelvic actinomycosis: case report. JSM Clin Case Rep.

[5] Lee YK, Bae JM, Park YJ, Park SY, Jung SY (2008). Pelvic actinomycosis with hydronephrosis and colon stricture simulating an advanced ovarian cancer. J Gynecol Oncol.

